# Investigation on the processing and improving the cleavage efficiency of furin cleavage sites in *Pichia pastoris*

**DOI:** 10.1186/s12934-018-1020-x

**Published:** 2018-11-08

**Authors:** Yide Huang, Yanyu Long, Suhuan Li, Ting Lin, Jingwen Wu, Yafei Zhang, Yao Lin

**Affiliations:** 1 Provincial University Key Laboratory of Cellular Stress Response and Metabolic Regulation, College of Life Sciences, Fujian Normal University, Fuzhou, 350007 China; 2 Key Laboratory of Optoelectronic Science and Technology for Medicine of Ministry of Education, Fujian Normal University, Fuzhou, 350007 China

**Keywords:** Furin, *Pichia pastoris*, Furin cleavage site, Proprotein convertase, Kex2, YPS1

## Abstract

**Background:**

Proprotein convertase furin is responsible for the processing of a wide variety of precursors consisted of signal peptide, propeptide and mature peptide in mammal. Many precursors processed by furin have important physiological functions and can be recombinantly expressed in *Pichia pastoris* expression system for research, pharmaceutical and vaccine applications. However, it is not clear whether the furin cleavage sites between the propeptide and mature peptide can be properly processed in *P. pastoris*, bringing uncertainty for proper expression of the coding DNA sequences of furin precursors containing the propeptides and mature peptides.

**Results:**

In this study, we evaluated the ability of *P. pastoris* to process furin cleavage sites and how to improve the cleavage efficiencies of furin cleavage sites in *P. pastoris*. The results showed that *P. pastoris* can process furin cleavage sites but the cleavage efficiencies are not high. Arg residue at position P1 or P4 in furin cleavage sites significantly affect cleavage efficiency in *P. pastoris*. Kex2 protease, but not YPS1, in *P. pastoris* is responsible for processing furin cleavage sites. Heterologous expression of furin or overexpression of Kex2 in *P. pastoris* effectively increased cleavage efficiencies of furin cleavage sites.

**Conclusions:**

Our investigation on the processing of furin cleavage sites provides important information for recombinant expression of furin precursors in *P. pastoris*. Furin or Kex2 overexpressing strains may be good choices for expressing precursors processed by furin in *P. pastoris*.

## Background

Many important peptides or proteins are synthesized intracellularly as higher-molecular-mass inactive precursors consisted of signal peptide, propeptide and mature peptide in eukaryotes from yeasts to mammals [1, 2]. Release of propeptide from inactive precursor proteins are proteolyzed by proprotein convertases within the secretory pathway, leading to the production of biologically active proteins or peptides. The product of the *KEX2* gene, Kex2 [also known as kexin (EC 3.4.21.61)], was the first identified proprotein convertase involved in the processing of α-mating factor and killer toxin precursors in *Saccharomyces cerevisiae*. Kex2 belonging to the subtilisin family has a catalytic domain similar to bacterial serine proteases and cleaves its precursors at paired basic residues [3]. Proprotein convertase furin, encoded by the *fur* gene, is the homologue of Kex2 in mammal [4]. Furin is expressed as a 794 amino acid precursor and rapidly converted into active mature form by two intramolecular autocatalytic cleavage (the first cleavage site, Arg-Ala-Lys-Arg^107^; the second cleavage site, Arg-Gly-Val-Thr-Lys-Arg^75^) [5]. The structure of furin includes a signal peptide, a propeptide, a subtilisin-like catalytic domain, a Homo B domain, a Cys-rich domain, a transmembrane domain and a cytoplasmic domain. Furin is capable of processing a wide variety of precursors including growth factors, hormones, neuropeptides, transcription factors, plasma proteins, receptors, viral envelope proteins, matrix metalloproteinases and bacterial exotoxins [1, 6]. Many substrates of furin have important physiological functions and possess great clinical values. It is attractive for the pharmaceutical industry to produce these substrates via recombinant protein expression systems.

*Pichia pastoris*, reclassified as *Komagataella phaffi* [7], has become a successful host organism for production of recombinant proteins due to the benefits of high cell density cultivation, the simplicity of genetic manipulation, growth on inexpensive media, efficient secretory capabilities with a low level of endogenous protein secretion, the strong and tightly inducible *AOX1* promoter and the ability of post-translational modifications to proteins [8, 9]. Currently, over 1000 proteins have been produced in *P. pastoris* [10]. In our previous studies, we heterologously expressed two furin substrates using the DNA sequences encoding the mature peptides in *P. pastoris*. But the recombinant proteins couldn’t form the proper native structures [11, 12]. Several studies have found that the propeptides in some precursors are important for the production of active mature peptides. The propeptides can act as intramolecular chaperones to assist the proper folding of mature peptides such as activin A and transforming growth factor-beta 1 (TGF-β1) [13], bone morphogenetic protein-4 (BMP-4) [14] and cathepsin L [15], or stabilize the mature peptides to increase the their transport from ER to Golgi [16, 17]. Therefore, co-expression of propeptide and mature peptide sequence may provide a strategy for efficient production of biologically active proteins processed by furin in *P. pastoris*. However, it is not clear whether the furin cleavage sites between the propeptide and mature peptide can be properly processed in *P. pastoris*, bringing uncertainty for proper expression of the DNA sequences of furin substrates containing the propeptides and mature peptides.

In this study, we used reporter proteins containing furin cleavage sites between the propeptide and mature peptide to systematically evaluate the ability of *P. pastoris* GS115 on processing furin cleavage sites. Our study will shed light on how to improve the efficiencies of producing biological active furin substrates in *P. pastoris*.

## Results and discussion

### Furin cleavage sites can be cleaved in *Pichia pastoris*

**Fig. 1:**
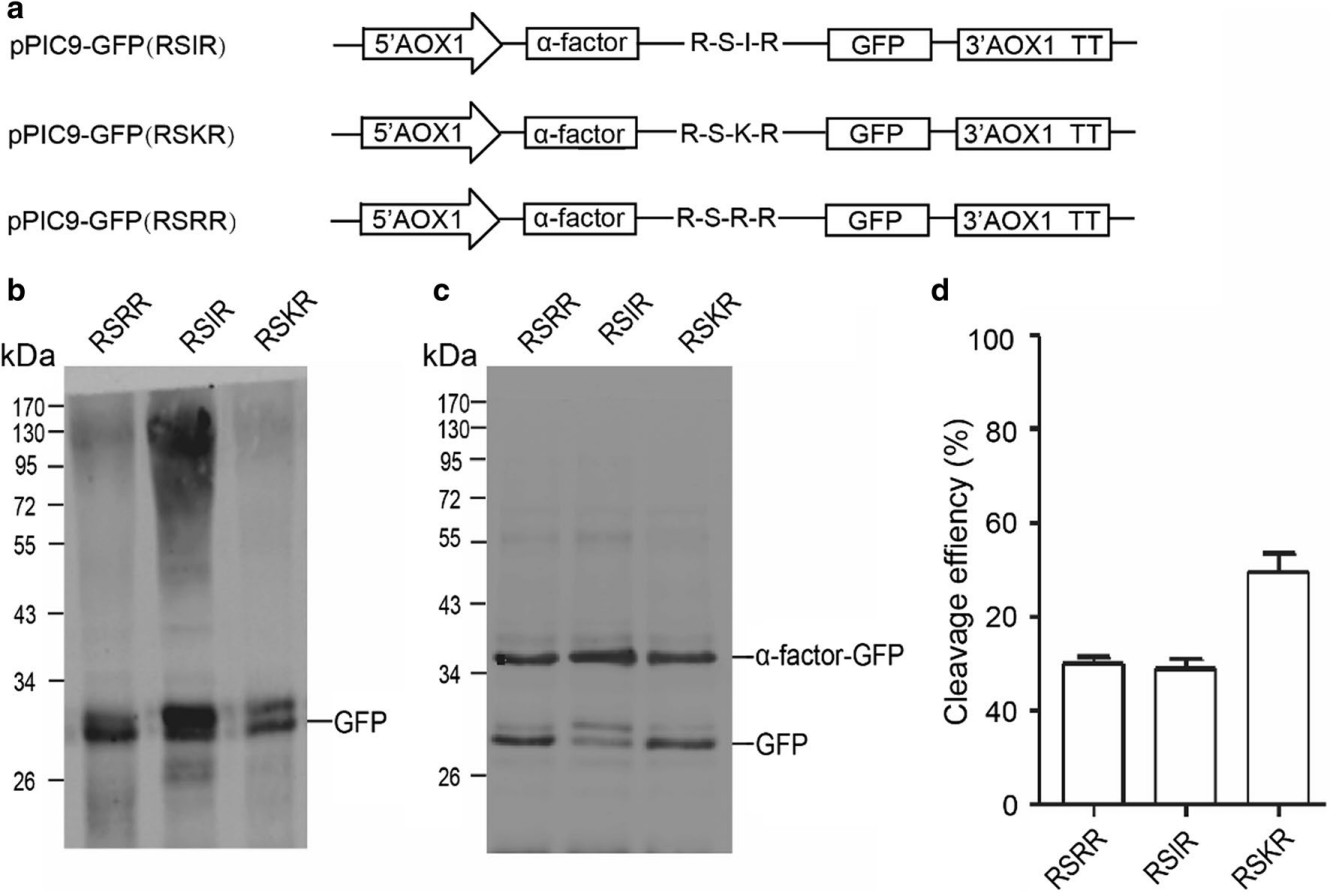
Cleavage of furin cleavage site in *Pichia pastoris*. **a** Schematic diagram of expression vectors using in this study. **b** Western blot of expressed products using anti-GFP antibody, the culture supernatants didn’t be treated with Endo Hf endoglycosidase which cleaves the chitobiose core of high mannose from N-linked glycoprotein. **c** Western blot of expressed products using anti-GFP antibody, the culture supernatants were treated with Endo Hf to show α-factor-GFP fusion protein after high mannose chains were removed from α-factor peptide. **d** Quantitative analysis of the western blot results, compared GFP with GFP and α-factor-GFP

In our previous study, we examined all reported precursors processed by furin and found there were three main cleavage sites between propeptides and mature peptides: Arg-Xaa-Lys-Arg (41%), Arg-Xaa-Arg-Arg (31.5%) and Arg-Xaa-Xaa-Arg (11%) (where Xaa is any amino acid) [18]. In order to determine if mammalian furin cleavage sites can be cleaved in *P. pastoris*, three reporter vectors containing different furin cleavage sites (Arg-Ser-Lys-Arg, Arg-Ser-Arg-Arg or Arg-Ser-Ile-Arg) between α-factor propeptide and GFP were constructed (Fig. 1a). The reporter vectors were introduced into *P. pastoris* GS115 by electroporation and the secreted proteins were subject to western blotting detection. A band indicating the cleaved GFP (about 27 kDa) can only be detected if furin cleavage sites could be cleaved in *P. pastoris*. The values of cleaved GFP band intensities divided by the total intensities of cleaved GFP band plus α-factor-GFP fusion protein band were regarded as the cleavage efficiency. As shown in Fig. 1b, GFP proteins in the supernatants were detected at expected molecular weight in all three recombinant strains, indicating furin cleavage sites could be cleaved to release the GFP. Considering the cleavage efficiency is unlikely to reach 100%, there should be some fusion proteins containing both α-factor and GFP. However, there were no bands detected at the expected 36 kDa, instead some smear bands at higher molecular weight were observed (Fig. 1b). We suspected that the smear bands were *N*-glycosylated fusion proteins of α-factor and GFP because there are three potential *N*-glycosylation motifs in α-factor peptide sequence. The culture supernatants were then treated with Endo Hf, which cleaves oligosaccharides from N-linked glycoproteins. The fusion proteins of α-factor and GFP were detected at 36 kDa after treatment (Fig. 1c). These results showed that all three furin cleavage sites can be partially cleaved in *P. pastoris*. The cleavage efficiency of Arg-Ser-Lys-Arg motif was the highest among the three furin cleavage motifs (Fig. 1d).

**Fig. 2:**
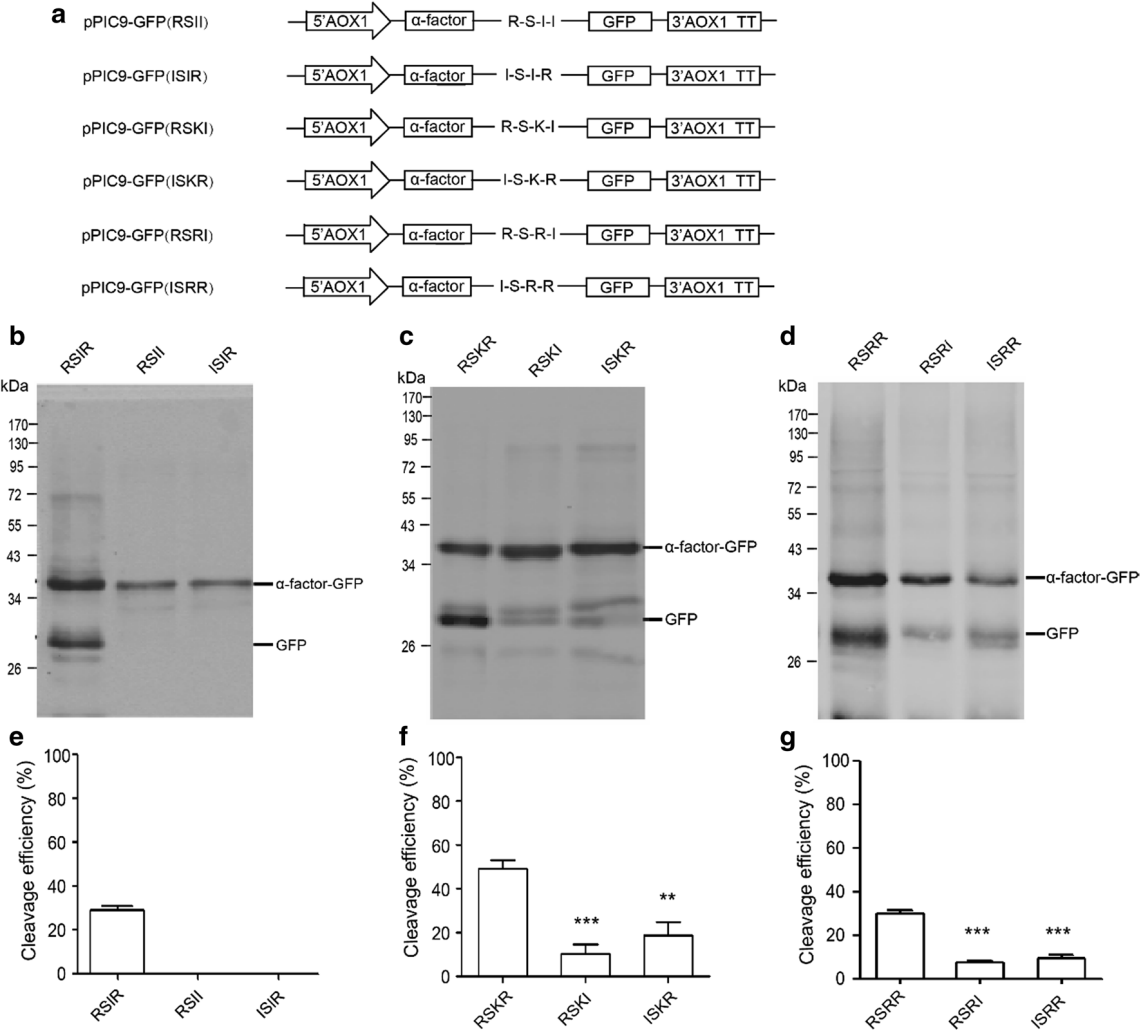
Arg residue at position P1 or P4 in furin cleavage sites effects on cleavage efficiency in *Pichia pastoris*. **a** Schematic diagram of expression vectors mutated furin cleavage sites from Arg to Ile at P1 or P4 position. **b**–**d** Western blot results of expressed products with three different furin cleavage sites and their corresponding mutants. The western blot samples were treated with Endo Hf. **e**–**g** Quantitative analysis of the western blot results. **p < 0.01, ***p < 0.001

Arg residues at position P1 and P4 in the cleavage site are essential for cleavage by furin in mammals [19, 20]. In order to evaluate the site preference of the proteases in *P. pastoris*, reporter vectors containing mutation of Arg at P1 or P4 were constructed (Fig. 2a). When Arg residue at P1 or P4 in Arg-Ser-Ile-Arg motif was mutated into Ile, the cleavage was completely lost in *P. pastoris* (Fig. 2b, e). Mutation of Arg into Ile at P1 or P4 in both Arg-Ser-Lys-Arg and Arg-Ser-Arg-Arg motifs significantly decreased the cleavage (Fig. 2c, d, f, g). Compared with Arg-Ser-Ile-Arg, Arg-Ser-Lys-Arg and Arg-Ser-Arg-Arg motifs contain one more basic amino acid. After mutation of Arg at position P1 or P4 into Ile, there were still two basic amino acids in Arg-Ser-Lys-Arg and Arg-Ser-Arg-Arg motifs left, suggesting two basic amino acids are sufficient furin cleavage sites processing in *P. pastoris*. Moreover, the two basic amino acids do not have to be paired and can be separated by one or two other amino acids. The cleavage efficiencies of Arg-Ser-Ile-Arg motif (29%) and Arg-Ser-Arg-Arg motif (29.7%), were similar, which were about two times lower than that of the Arg-Ser-Lys-Arg motif (49.3%). Suggesting it is more favorable for furin cleavage site processing in *P. pastoris* if the P2 position is Lysine. The Arg-Xaa-Xaa-Arg motif is also cleaved by furin in mammal, but the cleavage efficiency is tenfold lower than that of Arg-Xaa-(Lys/Arg)-Arg motif [21–23]. The difference in the cleavage efficiency towards Arg-Xaa-Xaa-Arg motif between *P. pastoris* and mammal suggested the proteases in *P. pastoris* is more flexible than furin.

At P1 position, both furin and *Saccharomyces cerevisiae* Kex2 exhibit extremely stringent specificity for Arg. Studies on furin substrate specificity have also shown that replacement of P1 Arg results in undetectable cleavage [24]. Substitution with similar positive charge amino acid, such as Lys or ornithine, at P1 position in *Saccharomyces cerevisiae* Kex2 peptidyl-MCA substrates also resulted in an approximately 100-fold drop in k_cat_/K_M_ relative to the canonical substrate sequence [25]. The cleavage efficiencies of all three furin processing sites were also significantly decreased when Arg residue at position P1 was mutated into Ile. For Arg-Ser-Ile-Arg motif, the cleavage was completely abolished. For Arg-Ser-Arg-Arg and Arg-Ser-Lys-Arg motifs, the cleavage efficiencies were greatly decreased. Our results showed that Arg residue at P1 is essential for cleavage in *P. pastoris*. For *Saccharomyces cerevisiae* Kex2, most of its selectivity towards substrates arises through interactions at position P1 and P2 [25, 26]. In *P. pastoris*, the amino acid at P2 seems to be less important if there is Arg at P4, suggesting that the selectivity of protease to substrates in *P. pastoris* is more like furin which generates most of its selectivity through interactions of Arg at P1 and P4.

### Kex2, but not YSP1, is responsible for processing the furin cleavage site in *Pichia pastoris*

**Fig. 3:**
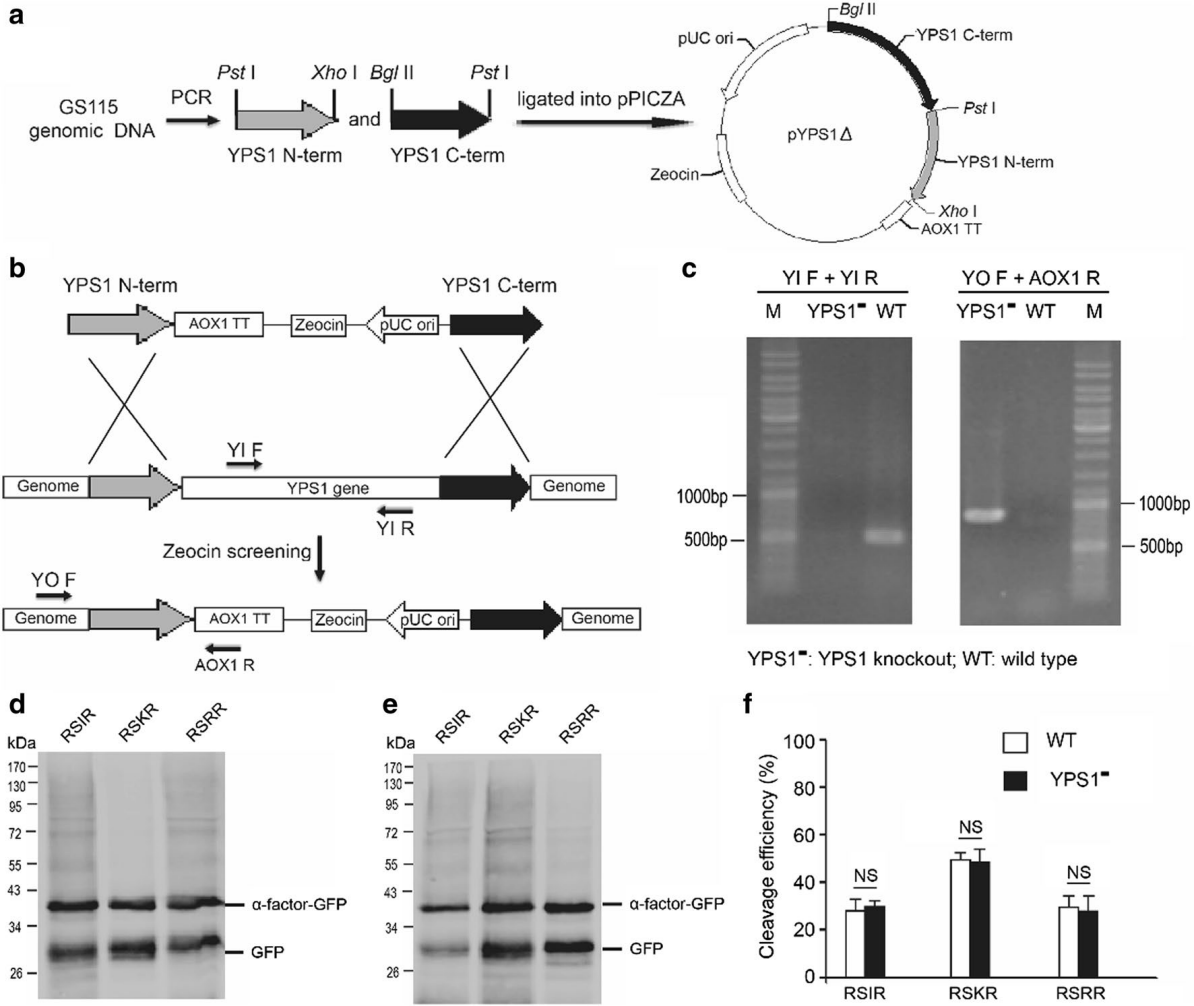
YPS1 doesn’t involve in processing the furin cleavage site in *Pichia pastoris*. **a** Schematic diagram for constructing a disruption vector with left homology arm and right homology arm of *YPS1* gene. **b** Schematic diagram for knockout of *YPS1* gene by homologous recombination. Zeocin can be used for selection of *YPS1* knockout strains. Two pairs primers, as shown in figure, were designed to confirm the disrupted strains. **c** Genomic PCR of *YSP1* disrupted and wild type strains using two pairs primers (YI F, YI R and YO F, AOX1 R). A band can be amplified by YI F and YI R primers only if *YPS1* gene doesn’t be deleted, and a band can be amplified by YO F and AOX1 primers if *YPS1* gene is successfully removed by the disruption construct. **d** Western blot results of wild type strains. The western blot samples were treated with Endo Hf. **e** Western blot results of *YPS1* disruption strains. The western blot samples were treated with Endo Hf. **f** Quantitative analysis of the western blot results. *NS* not statistically significant

Our results indicated that furin cleavage sites can be processed in *P. pastoris*, but the exact proteases responsible for this processing are unclear. Genome sequencing of *P. pastoris* GS115 revealed the presence of a family of glycosylphosphatidylinositol (GPI)-linked aspartyl proteases named yapsins [27]. YPS1 (yapsin 1, previously named as Yap3; EC 3.4.23.41), a member of yapsins, was shown to cleave its substrates at mono- and paired basic amino acid residues [28, 29]. Since there are mono- or paired basic amino acid residues in the three furin cleavage motifs, we first speculated that YSP1 is involved in the proteolytic cleavage of furin cleavage sites. We constructed the disruption plasmid pYPS1Δ (Fig. 3a), and *YPS1* gene was disrupted by insertion of the zeocin-selectable plasmid pYPS1Δ (Fig. 3b). Correct disruption of *YPS1* gene was confirmed by PCR using two pairs of specific primers (Fig. 3c). The cleavage efficiencies in three furin cleavage sites were similar between wild type and the disruptants (Fig. 3d–f), indicating that YPS1 is not involved in the processing of furin cleavage sites in *P. pastoris*.

**Fig. 4:**
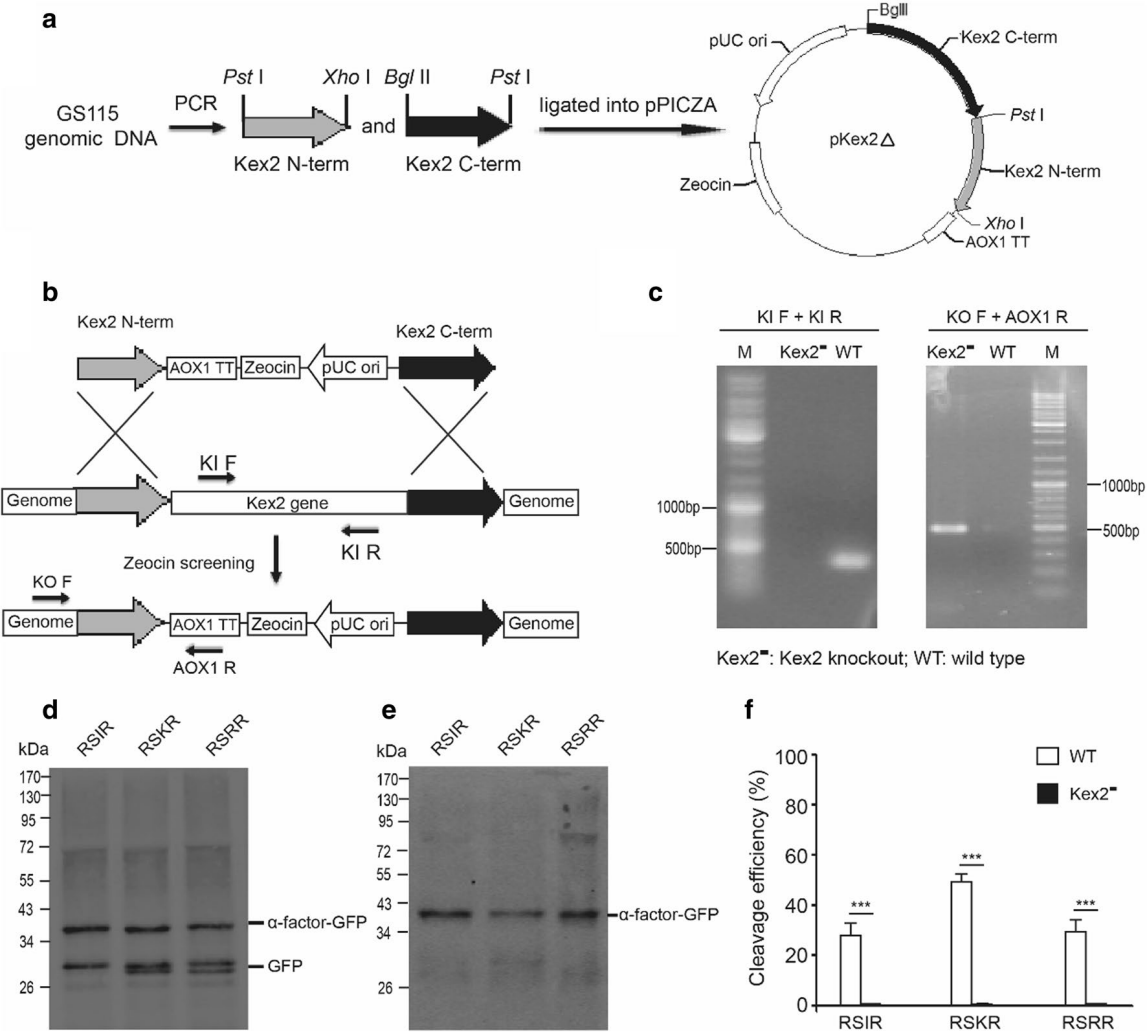
Kex2 involved in processing the furin cleavage site in *Pichia pastoris*. **a** Schematic diagram for constructing a targeting vector with left homology arm and right homology arm of *Kex2* gene. **b** Schematic diagram for knockout of *Kex2* gene by homologous recombination. Zeocin can be used for selection of *Kex2* disruption strains. Two pairs primers, as shown in figure, were designed to confirm the disruption strains. **c** Genomic PCR of *Kex2* disruption and wild type strains using two pairs primers (YI F, YI R and YO F, AOX1 R). A band can be amplified by YI F and YI R primers only if *Kex2* gene doesn’t be deleted, and a band can be amplified by YO F and AOX1 primers if *Kex2* gene is successfully removed by the targeting construct. **d** Western blot results of wild type strains. The western blot samples were treated with Endo Hf. **e** Western blot results of *Kex2* disruption strains. The western blot samples were treated with Endo Hf. **f** Quantitative analysis of the western blot results. ***p < 0.001

In addition to YPS1, we also investigated the effect of Kex2 protease on furin cleavage site processing. Kex2 is involved in processing α-mating pheromone and killer toxin precursors by limited proteolysis at the carboxyl side of the paired basic amino acid residues [4, 30]. Biochemical analysis has also shown that Kex2 exhibits high activity toward substrates with Lys-Arg or Arg-Arg cleavage motifs [25, 31]. The *Kex2* gene was disrupted by homologous recombination using a similar strategy for disrupting *YPS1* gene (Fig. 4a–c). All of three furin cleavage sites were not cleaved in Kex2 disrupted strains (Fig. 4d, e), indicating Kex2 protease is responsible for the processing of furin cleavage sites in *P. pastoris*.

### Heterologous expression of furin or overexpression of Kex2 significantly increased cleavage efficiency of furin cleavage sites in *Pichia pastoris*

**Fig. 5:**
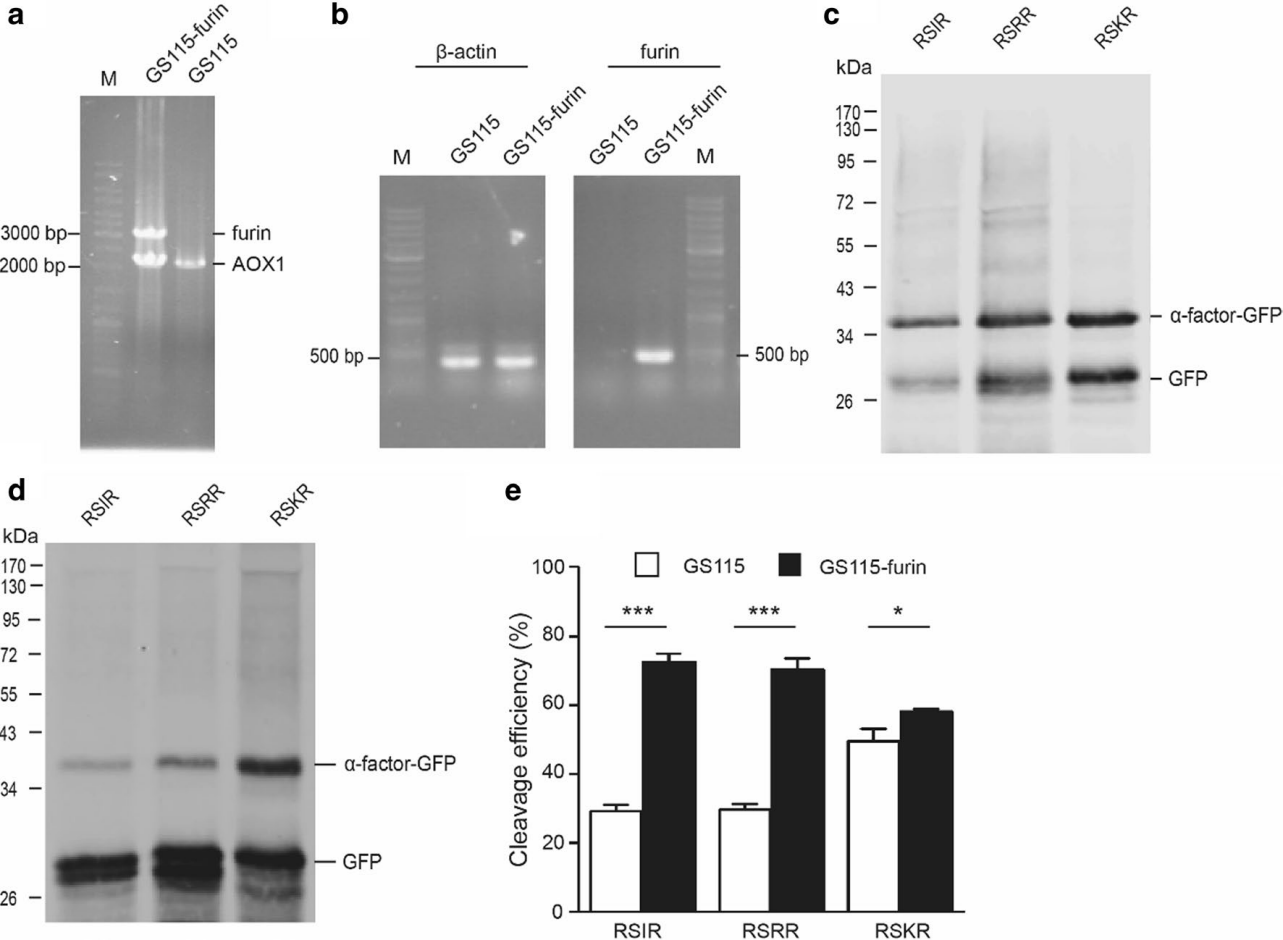
Heterologous expression of furin increased cleavage efficiency of furin cleavage sites in *Pichia pastoris*. **a** Genomic PCR for analyzing integration of furin gene into GS115 genome using AOX1 general primers. **b** Expression analysis of integrated furin gene in *Pichia pastoris* GS115 by RT-PCR with β-actin and furin specific primers. **c** Western blot results of GS115 strains. The western blot samples were treated with Endo Hf. **d** Western blot results of GS115-furin strains. The western blot samples were treated with Endo Hf. **e** Quantitative western blot analysis of cleavage efficiency of three furin cleavage sites. *p < 0.05, ***p < 0.001

**Fig. 6:**
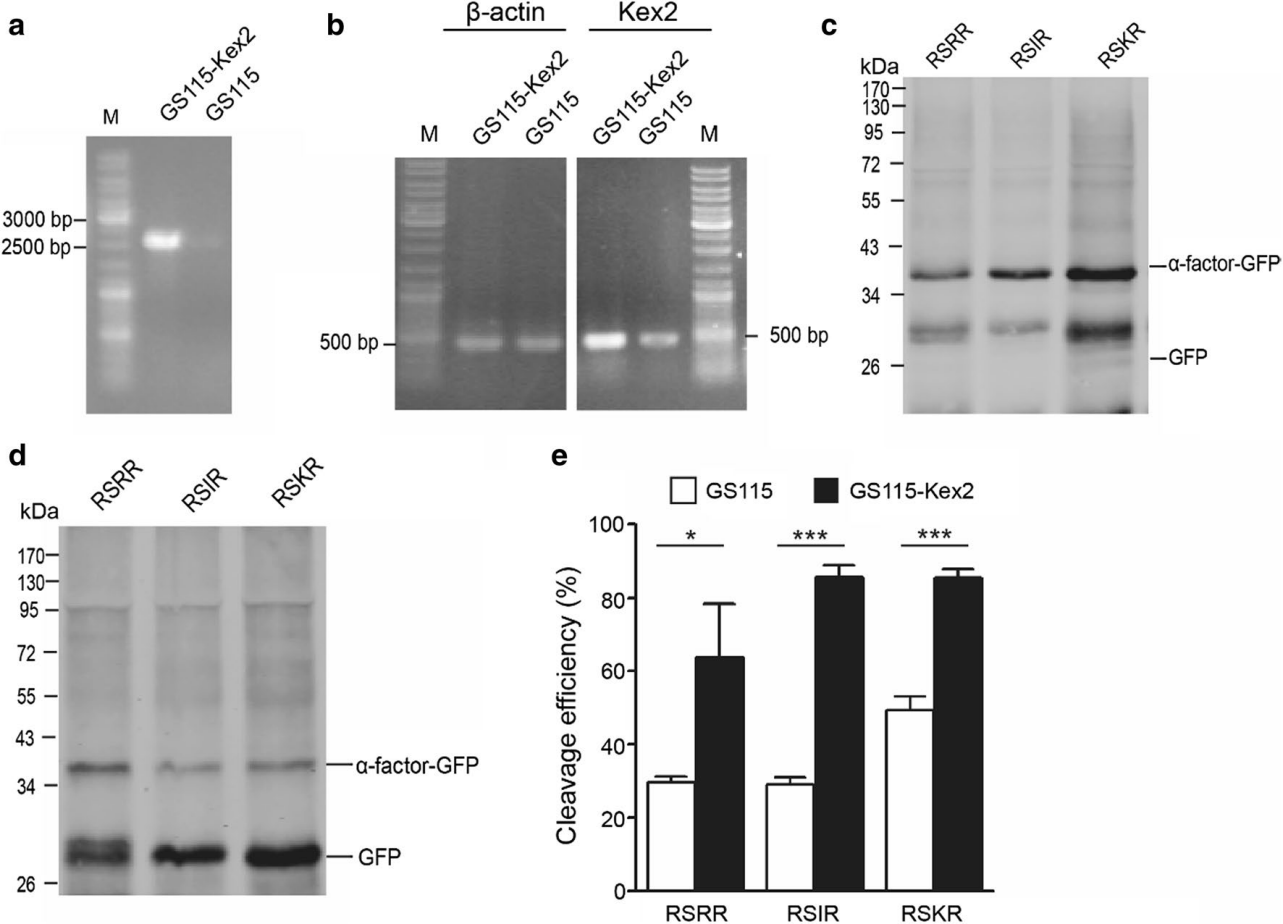
Overexpression of Kex2 increased cleavage efficiency of furin cleavage sites in *Pichia pastoris*. **a** Genomic PCR for analyzing integration of *Kex2* gene into GS115 genome using 5′ GAP general upstream and Kex2 specific downstream primers. **b** Kex2 overexpression analysis in GS115-Kex2 strains by RT-PCR. Expression of β-actin was used as control. **c** Western blot results of GS115 strains. The western blot samples were treated with Endo Hf. **d** Western blot results of GS115-Kex2 strains. The western blot samples were treated with Endo Hf. **e** Quantitative western blot analysis of cleavage efficiency of three furin cleavage sites in GS115 and GS115-Kex2 strains. *p < 0.05, ***p < 0.001

Although endogenous Kex2 in *P. pastoris* can process furin cleavage sites, the efficiency is not high. If the DNA sequence of furin substrates were expressed in *P. pastoris*, the production of the mature peptides will be limited due to low cleavage efficacy. It will be useful to generate an engineered *P. pastoris* strain that is capable of efficient processing furin cleavage sites. Based on our above results, *furin* or *Kex2* gene were integrated into *P. pastoris* genome. Cleavage efficiencies in three furin cleavage sites were evaluated in GS115-furin and GS115-Kex2 strains. As expected, the cleavage efficiencies in all three furin cleavage sites were significantly increased in both GS115-furin and GS115-Kex2 strains (Figs. 5 and 6), suggesting these two engineered strains can be applied for efficient production of furin substrates.

Recently, studies on ligase derived from fungus showed that co-expression of propeptide and mature peptide in *P. pastoris* helped mature peptide form the proper protein conformation [32, 33]. A large number of propeptide of precursor proteins have been identified as intramolecular chaperones (IMCs) and assist the proper folding of mature peptides. Propeptides of precursors processed by furin were also found to be critical in correct protein folding of mature peptides [13, 14]. Further researches are needed to verify whether the expression of the furin substrate DNA sequence consisted of both the propeptide and mature peptide in *P. pastoris* help enhance to activity of the mature peptide. If this strategy proves to produce more active furin substates in comparison with direct expression of the mature peptide DNA sequence, our GS115-furin and GS115-Kex2 strains may be good choices for expressing precursors processed by furin in *P. pastoris*.

## Conclusions

In this study we showed that Kex2, but not YPS1, in *P. pastoris* is capable of processing furin cleavage sites and the Arg residue at position P1 or P4 of furin cleavage sites significantly affect the cleavage efficiency. Overexpression of both furin or Kex2 in *P. pastoris* effectively increased the processing efficiency of furin cleavage sites, providing an attractive direction for efficiently express bioactive furin substrates in *P. pastoris*.

## Methods

### Enzymes and reagents

Restriction enzymes, T4 DNA ligase, *Taq* DNA polymerase, polymerase chain reaction (PCR) reagent, Prime STAR polymerase, and PCR reagent were obtained from Takara Bio Inc. The Endo Hf was purchased from New England Biolabs, Inc. SDS-PAGE Protein Marker was supplied by Thermo Fisher Scientific. Primers were all synthesized at Sangon Biotech (China). PCR Purification Kit was from Promega Corporation. Plasmid Mini Kit and Point mutation kit were from TransGen Biotech (China). All other chemicals used were acquired from Sigma Aldrich Chemicals P Ltd. and Sinopharm Chemical Reagent (China).

### Strains and plasmids

*Escherichia coli* Top10F’ was used as the cloning host. *P. pastoris* GS115(*his 4*^−^) was used as the expression host. Plasmid pPIC9 and pGAPZB were used as gene expression vectors. All of them were purchased from Thermo Fisher Scientific (Invitrogen). The recombinant plasmid pPIC9-GFP with different furin cleavage sites, YPS1 disruption vector pYPS1Δ, Kex2 disruption vector pKex2Δ, pGAPZB-Kex2 and pGAPZB-furin were constructed and stored in our lab.

### Preparation of DNA constructs

All primer sequences using in this study were listed in Table 1. To construct plasmid pPIC9-GFP(RSIR), GFP was amplified from pEGFP-N1 using a pair of primer, upstream primer with *Xho*I site and Arg-Ser-Ile-Arg(RSIR) motif sequence, and downstream primer with *Eco*RI site. The amplified fragment was cloned into pPIC9 vector at *Xho*I and *Eco*RI sites. The pPIC9-GFP(RSII), pPIC9-GFP(ISIR), pPIC9-GFP(RSRR) and pPIC9-GFP(RSKR) were constructed through mutating RSIR motif of pPIC9-GFP(RSIR) plasmid using site-directed mutagenesis method, directly. pPIC9-GFP(RSRI) and pPIC9-GFP(ISRR) were generated using pPIC9-GFP(RSRR) as template by site-directed PCR mutagenesis. Both of pPIC9-GFP(RSKI) and pPIC9-GFP(ISKR) were generated using pPIC9-GFP(RSKR) as template.

**Table 1:** Primers using in this study

Description	Primer name	Primer sequence (5′–3′)	Restriction site
Preparation for expression vectors			
pPIC9-GFP(RSIR)	RSIR F	CCGCTCGAGAGATCTATCAGAGTGAGCAAGGGCGAG	*Xho*I
	RSIR R	CCGGAATTCTTACTTGTACAGCTCGTC	*Eco*RI
pPIC9-GFP(RSII)	RSII F	CTCTCGAGAGATCTATAATAGTGAGCAAGGG	
	RSII R	CCCTTGCTCACTATTATAGATCTCTCGAGAG	
pPIC9-GFP(ISIR)	ISIR F	CTCTCGAGATATCTATAAGAGTGAGCAAGGG	
	ISIR R	CCCTTGCTCACTCTTATAGATATCTCGAGAG	
pPIC9-GFP(RSRR)	RSRR F	CTCTCGAGAGATCTAGAAGAGTGAGCAAGGG	
	RSRR R	CCCTTGCTCACTCTTCTAGATCTCTCGAGAG	
pPIC9-GFP(RSRI)	RSRI F	CTCTCGAGAGATCTAGAATAGTGAGCAAGGG	
	RSRI R	CCCTTGCTCACTATTCTAGATCTCTCGAGAG	
pPIC9-GFP(ISRR)	ISRR F	CTCTCGAGATATCTAGAAGAGTGAGCAAGGG	
	ISRR F	CCCTTGCTCACTCTTCTAGATATCTCGAGAG	
pPIC9-GFP(RSKR)	RSKR F	CTCTCGAGAGATCTAAGAGAGTGAGCAAGGG	
	RSKR R	CCCTTGCTCACTCTCTTAGATCTCTCGAGAG	
pPIC9-GFP(RSKI)	RSKI F	CTCTCGAGAGATCTAAGATAGTGAGCAAGGG	
	RSKI R	CCCTTGCTCACTCTCTTAGATATCTCGAGAG	
pPIC9-GFP(ISKR)	ISKR F	CTCTCGAGATATCTAAGAGAGTGAGCAAGGG	
	ISKR R	CCCTTGCTCACTATCTTAGATCTCTCGAGAG	
pGAPZ B-Kex2	Kex2 F	CCGGAATTCCGGATGTATTTGCCAGCACTT	*Eco*RI
	Kex2 R	CCGCTCGAGCGGTTACAATGCCGCACGTTT	*Xho*I
pGAPZ B-furin	Furin F	CCGGAATTCCGGATGGAGCTGAGGCCCTGG	*Eco*RI
	Furin R	CCGCTCGAGTTAGAGGGCGCTCTGGTCTT	*Xho*I
Preparation for disruption vectors			
pYPS1Δ	Left arm F	TGCACTGCAGTAGCCGTTCCCGCGTGAAGA	*Pst*I
	Left arm R	CCGCTCGAGCGGACTAGCATATGTGGATTCTAG	*Xho*I
	Right arm F	GGAAGATCTTCCTCCATCCTCTTTGGAGGTGTG	*Bgl*II
	Right arm R	TGCACTGCAGTGCACTATACTATACACACG	*Pst*I
pKex2Δ	Left arm F	TGCACTGCAGTGCAATGTATTTGCCAGCAC	*Pst*I
	Left arm R	CCGCTCGAGCGGTGGTGGATGAAGCCCTTTAAT	*Xho*II
	Right arm F	GGAAGATCTTCCACGGATATGGCAAGATCGATG	*Bgl*II
	Right arm R	TGCACTGCAGTGCATTACAATGCCGCACGTTTGGG	*Pas*tI
Identification for disrupted strains			
GS115-ΔYps 1	YI F	CAGTATGACAATTTGCCAGC	
	YI R	TCCCCCGGTGTAATATGTTG	
	YO F	GGACTTCAGCGTTCTGAGGG	
	AOX1 R	GCAAATGGCATTCTGACATCC	
GS115-ΔKex 2	KI F	GGCTTGCTCTGCTGTGATGA	
	KI R	AGATCTTTGGCCTCCTGGG	
	KO F	CACCTGAAACTTAATACTCT	
	AOX1 R	GCAAATGGCATTCTGACATCC	
Identification for GS115-furin and GS115-Kex2 strains			
GS115-furin	AOX1 F	GACTGGTTCCAATTGACAAGC	
	AOX1 R	GCAAATGGCATTCTGACATCC	
GS115-Kex2	GAP F	GTCCCTATTTCAATCAATTGAA	
	Kex2 R	GCCTCCTGGGTCAATTCATA	
RT-PCR			
β-actin	β-actin F	CTCCAATGAACCCAAAGTCCAAC	
	β-actin R	GACAAAACGGCCTGAATAGAAAC	
Furin	RT-furin F	GGCATTGTGGTCTCCATTCT	
	RT-furin R	GCAGTTGCAGCTGTCATGTT	
Kex2	RT-Kex2 F	GGCATTGTGGTCTCCATTCT	
	RT-Kex2 R	GCAGTTGCAGCTGTCATGTT	

To construct *YPS1* and *Kex2* gene disruption vectors, The *P. pastoris* GS115 genome was extracted and used as template to amplify the left homology arm and right homology arm with two pairs of specific primers to *YPS1* or *Kex2* gene. The left arm fragment contains *Pst*I site at 5′ end and *Xho*I at 3′ end, and the right arm fragment contains *Bgl*II site at 5′ end and *Pst*I at 3′ end. pPICZα A was used as the backbone to generate the disruption vectors. Both of pPICZα A and the left arm fragment were digested with the *Pst*I and *Xho*I restriction endonucleases and ligated to produce an intermediate plasmid, The intermediate plasmid then was digested with the *Pst*I and *Bgl*II restriction endonucleases. The right arm fragment digested with the same restriction endonucleases was inserted into the intermediate plasmid at the *Pst*I and *Bgl*II sites to produce the disruption vector.

In order to overexpress Kex2 protease in *P. pastoris* GS115, *Kex2* gene was amplified from *P. pastoris* GS115 genome by PCR and cloned into pGAPZ B expression vector at *Eco*RI and *Xho*I sites. To heterologous expression of furin proprotein convertase in GS115, *furin* cDNA was amplified by PCR using pGEM-furin plasmid (a gift kindly provided by professor Gary Thomas from Department of Cell and Developmental Biology, Oregon Health and Science University, School of Medicine, Portland, USA) as the template, and cloned into pGAPZ B expression vector at *Eco*RI and *Xho*I sites.

### Electroporation

The recombinant plasmids pPIC9-GFP(RSIR), pPIC9-GFP(RSRR), pPIC9-GFP(RSKR), pPIC9-GFP(ISIR), pPIC9-GFP(RSII), pPIC9-GFP(ISRR), pPIC9-GFP(RSRI), pPIC9-GFP(ISKR) and pPIC9-GFP(RSKI) were linearized with *Sal*I restriction endonuclease. pKex2Δ, pYPS1Δ were linearized with *Pst*I restriction endonuclease. pGAPZ B-Kex2 and pGAPZ B-furin were linearized with *Avr*II restriction endonuclease. 10 μg linearized plasmid DNA was transformed into *P. pastoris* GS115 competent cells by electroporation (1.5 kV, 25 µF, 200 Ω) using a Bio-Rad Gene Pulser (Hercules, USA). The transformed cells were screened on MD (0.34% YNB, 4 × 10–5% biotin, 1% dextrose, and 1.5% agar) or YPD (1% yeast extract, 2% peptone, 2% dextrose and 1.5% agar) in the presence of 25 µg/ml Zeocin (Invitrogen) plates.

### Expression of protein in shaking flasks

Colonies were picked into BMGY medium (1% yeast extract, 2% peptone, 0.34% YNB, 4 × 10^−5^ % biotin, 0.5% methanol, 100 mM potassium phosphate, pH 6.0,) and incubated in an orbital shaker at 220 rpm for 24 h until the OD_600 nm_ reached 2–5. The supernatant was discarded by centrifugation at 1500 rcf for 10 min at room temperature, and the cells were resuspended in 10 ml BMMY medium (1% yeast extract; 2% peptone; 100 mM phosphate buffer, pH 6.0; 1.34% yeast nitrogen base, 4 × 10^−5^ % biotin; and 1% methanol) to a final OD_600 nm_ = 1 in 250 ml shaking flasks. Methanol was added to induce expression of GFP every 24 h. After shaking in flasks for 72 h, the supernatant was harvested.

### Deglycosylation by Endo Hf

Due to the presence of glycosylation sites in the α-factor peptide, the expressed α-factor-GFP fusion protein was glycosylated. Endo Hf (New England Biolabs) was used to remove *N*-glycan of α-factor-GFP fusion protein. Briefly, the culture supernatant was boiled for 10 min in denatured buffer containing 0.4 M DTT and 0.5% SDS, and deglycosylation was then performed by treatment with Endo Hf at 37 °C for overnight according to the instructions of the manufacture. The buffer employed in these enzyme reactions was 50 mM sodium citrate (pH 5.5) for Endo Hf.

### Determination of the cleavage efficiency of furin cleavage sites in *Pichia pastoris* by western blotting

Cleavage efficiency to different furin cleavage sites in *P. pastoris* was validated by western blotting. The proteins in culture supernatant were separated by SDS-PAGE (12% gel), and then transferred onto a Protran nitrocellulose membrane (Signa Amersham) using a Mini Trans-Blot Cell (Bio-Rad). The membranes were blocked with 5% fat-free milk and incubated overnight at 4 °C with anti-GFP antibodies at 1:500 dilutions (Santa Cruz Biotechnology, Santa Cruz, CA, USA). IRDye 800CW goat anti-mouse immunoglobulin G (LI-COR Biosciences) was used as the secondary antibody at 1:1000 dilutions. Detection and quantification of proteins were performed using Odyssey imaging systems (LI-COR Biosciences). Cleavage efficiency was calculated by the following equation: cleavage efficiency = sum of cleaved GFP band intensities/sum of cleaved GFP and α-factor-GFP fusion protein band intensities. All the experiments were repeated at least three times independently.

### RT-PCR

Total RNA of Kex2 or furin knockin GS115 strains was obtained using Yeast RNAiso Kit (Takara Bio Inc.) and cDNA was synthesized from 500 ng of the total RNA using Reverse Transcription System (Takara Bio Inc.). The primer sequences used in this work are shown in Table 1. The PCR mixture was first heated to 95 °C for 5 min, then entering 30 cycles of 94 °C for 30 s, 55 °C for 30 s and 72 °C for 30 s. The PCR products were analyzed by 1.5% agarose gel electrophoresis with ethidium bromide staining.
